# The link between childhood traumatic events and the continuum of premenstrual disorders

**DOI:** 10.3389/fpsyt.2024.1443352

**Published:** 2024-10-09

**Authors:** Lindsay R. Standeven, Mira Bajaj, Kathleen McEvoy, Dalar Shirinian, Kristin Voegtline, Lauren M. Osborne, Jennifer L. Payne, Liisa Hantsoo

**Affiliations:** ^1^ Department of Psychiatry and Behavioral Sciences, Johns Hopkins University School of Medicine, Baltimore, MD, United States; ^2^ Johns Hopkins University School of Medicine, Baltimore, MD, United States; ^3^ Department of Mental Health, Johns Hopkins Bloomberg School of Public Health, Baltimore, MD, United States; ^4^ Department of Psychiatry, Valley Health System, Las Vegas, NV, United States; ^5^ Department of Obstetrics and Gynecology, Weill Cornell Medical College, New York, NY, United States

**Keywords:** childhood trauma, premenstrual syndrome, premenstrual exacerbation (PME), Premenstrual Dysphoric Disorder (PMDD), adverse childhood experiences, menstrual cycle

## Abstract

**Background:**

Premenstrual Syndrome (PMS) and Premenstrual Dysphoric Disorder (PMDD), collectively known as Premenstrual Disorders (PMDs), cause significant distress and functional impairment, and premenstrual exacerbation (PME) affects a large proportion of women with psychiatric diagnoses. Childhood trauma is one factor that may contribute to PMD/PME risk. This study examines the relationship between childhood trauma and PMDs, PME, and non-PMD psychiatric illness.

**Methods:**

This study is a secondary analysis of data from a prospective cohort. Participants completed self-assessments on childhood trauma using the Childhood Traumatic Event Scale (CTE-S) and on premenstrual symptoms using the Premenstrual Symptoms Screening Tool (PSST). Psychiatric diagnoses were assessed through structured clinical interviews. Participants were divided into four groups based on their PSST scores and psychiatric illness status: (1) Premenstrual Disorders (PMDs; moderate to severe PMS and PMDD), (2) PME, (3) psychiatric controls (PC; individuals with psychiatric illness but no significant premenstrual symptoms), and (4) healthy controls (HC; individuals with no psychiatric illness and no significant premenstrual symptoms). Statistical analyses, including ANOVA, Tukey’s HSD test, Fisher’s exact test, and logistic regression, were conducted to examine differences among the groups.

**Results:**

Data from 391 participants were analyzed. Participants with PME and PC reported a higher quantity and severity of childhood traumatic events compared to HCs (p <.05). There was a weak but significant correlation between childhood trauma and premenstrual symptom burden across all groups (R = .18, p <.001). Within-group analysis revealed moderate correlations between childhood trauma and premenstrual symptoms driven by the PMD group (R = .42, p = .01).

**Conclusions:**

The findings underscore the impact of childhood traumatic events on mental health and premenstrual symptoms and highlight the need for additional research to explore the underlying mechanisms linking childhood trauma to the continuum of premenstrual disorders, to improve the efficacy of trauma-focused interventions for affected individuals.

## Introduction

On average, women menstruate for 40 years of their lives, and approximately 80% experience physical and/or mood symptoms in the premenstrual week. While most women experience mild premenstrual symptoms that do not affect quality of life or functioning, an estimated 20-40% of women experience premenstrual syndrome (PMS) and 5-8% meet criteria for Premenstrual Dysphoric Disorder (PMDD). Premenstrual Syndrome (PMS) is characterized by recurrent physical and/or emotional symptoms occurring in the late luteal phase (roughly the week prior to the onset of menses); symptoms remit with the start of menstruation (1). Although these symptoms can affect daily functioning and quality of life, the affective symptoms that occur do not reach threshold for a depressive disorder, and some women have physical symptoms only. PMDD, on the other hand, is a mood disorder characterized by functional impairment secondary to affective changes in the late luteal phase. Collectively, these premenstrual syndromes (PMS and PMDD), often thought of as a continuum of Premenstrual Disorders (PMDs), contribute to significant physical and psychic burden across a woman’s reproductive lifespan.

In addition to the PMDs, there is Premenstrual Exacerbation (PME) of psychiatric disorders. PME occurs in women with psychiatric disorders who experience a significant worsening in psychiatric symptoms during the week preceding their menses. PME of affective disorders is common. Recent retrospective and prospective studies have indicated PME prevalence rates ranging from 33.7% to 68.5% in women with major depressive disorder and 44-68% among those with bipolar depression (2–7). PME is associated with increased morbidity of psychiatric illness, including decreased general functioning, shorter remission times, more severe symptoms, and higher rates of treatment resistance. Like the PMDs, PME is thought to involve abnormal sensitivity to ovarian hormone fluctuations, although specific mechanisms remain unclear and could vary based on primary diagnosis (2).

Numerous biopsychosocial factors have been proposed as potential contributors to PMDs, including genetic predisposition, hormone sensitivity, neurotransmitter alterations, and inflammatory processes (8–14). Amidst these potential contributors, the influence of psychosocial stress, particularly trauma or adverse childhood experiences (ACEs), has emerged as a contributor to the risk for and severity of PMDs (2, 3, 15). ACEs refer to potentially traumatic events or experiences occurring in childhood or adolescence that fall into one of three general categories and can include one or many of the following: abuse (physical, emotional or sexual), neglect (physical or emotional) and household challenges (substance abuse, mental illness, domestic violence, parental separation or divorce, incarceration) (15). Several studies have found an association between the number of ACEs and the development and severity of PMDs. In a large cross-sectional analysis of nearly 12,000 participants, Yang et al. (2022) found a positive linear association between the number of ACEs experienced and probability of PMDs, with those experiencing >4 ACEs having a higher likelihood of having PMDD (7). Another study observed a similar correlation where the number and severity of premenstrual symptoms increased with increasing levels of childhood trauma (16). In a recent study, more childhood adversity was associated with increasing negative affect and greater stress response (as measured by cortisol) as patients progressed from the follicular to the luteal phase among women with PMDD (17).

Other research has focused on whether specific types of traumatic or adverse childhood experiences are associated with higher risk for or severity of PMDs. For example, some studies found a significant association between childhood emotional and physical abuse and later risk for PMDs (18–22). Yet other investigations pointed to sexual abuse during childhood and adolescence as posing the highest risk for PMDs, and particularly for PMDD (23). Conversely, others failed to find an association between sexual abuse and PMDs (24). Some studies found higher rates of PMDD among women with higher levels of childhood adversity independent of category (i.e. physical abuse, sexual abuse, emotional abuse, and neglect). Together, these conflicting results may stem from differences in study methodology, varying definitions of PMDs (i.e., whether specifically studying PMDD versus PMS), limited sample sizes, and a scarcity of studies focused on the stressor timing (childhood versus lifetime adversity) in relation to the onset or severity of PMDs.

Despite the known connection between childhood trauma and increased risk for various mental health conditions (25, 26), including PMDs, only a few studies have explored the relationship between childhood trauma or adversity and PME. One such study by Koci et al. (2007) assessed the impact of childhood sexual and physical abuse on subsequent symptoms of PMS and mood symptoms throughout the menstrual cycle and discovered that both types of abuse were elevated among women experiencing “premenstrual magnification” of mood symptoms and among women with persistently heightened mood symptoms across the menstrual cycle, irrespective of premenstrual changes (23). Although this study underscores a connection between trauma and the development of *both* mood symptoms and PME, its applicability is constrained by the absence of formal psychiatric evaluations.

To date, significant gaps persist in our understanding of the correlations between PMDs (as well as PME) and the timing (e.g. childhood exposure), type, and severity of traumatic events; thus the present study aims to assess these relationships within a cohort of women who underwent thorough psychiatric assessment and premenstrual symptom evaluation. We defined participant groups by their primary diagnosis and premenstrual symptoms (1) PMDs (2), PME (3), psychiatric controls (PC) (4), healthy controls (HC), and evaluated associations with the frequency, nature, and timing of childhood traumatic events. We hypothesized that individuals with PMDs would have more childhood trauma (in both quantity and severity) compared to those with PME, psychiatric illness alone, and controls.

## Methods

This is a secondary analysis of data from The Prospective Study of Pregnant Women, an observational cohort of pregnant individuals aged 18 and older with and without mood disorders at The Johns Hopkins University School of Medicine (27, 28). Exclusion criteria included current active suicidal ideation, medical instability, and active substance use disorders. Upon enrollment in the study, participants filled out a variety of study questionnaires, including demographic information, reproductive history including age of menarche, and self-assessments focusing on trauma and menstrual cycle symptoms: the Childhood Traumatic Event Scale (CTE-S), which gauges the history of childhood trauma, and the Premenstrual Symptoms Screening Tool (PSST), which collects retrospective assessments of mood and physical premenstrual symptoms. Past and current psychiatric diagnoses were determined with a Structured Clinical Interview for DSM-IV Disorders (SCID) (29) conducted by a trained research assistant and confirmed through clinical interview by a psychiatrist using DSM-IV criteria. Any discrepancy in diagnosis was reconciled through review by the study psychiatrists and research assistant. Data were collected and securely stored using the REDCap (Research Electronic Data Capture) tool hosted at Johns Hopkins University (30, 31). This study was approved by the Institutional Review Board of The Johns Hopkins University.

### Study measures

Childhood Traumatic Events Scale (CTE-S): The Childhood Traumatic Events Scale (CTE-S) is a self-report questionnaire used to assess exposure to different types of trauma prior to the age of 17. This scale (32) assesses whether participants experienced six different types of trauma: 1) death of a close friend or family member, 2) major upheaval between parents (divorce, separation, etc.), 3) traumatic sexual experience, 4) physical violence, 5) extreme illness or injury, 6) “other major upheaval.” For each type of trauma, participants are first asked whether they experienced it prior to the age of 17 or not. If they endorse experiencing it, they are asked further questions about their age at the time of trauma, the severity of how traumatic this event was (Likert scale 1 to 7), and how much they confided in others (Likert scale 1 to 7). For each participant, the sum of total number of traumatic events experienced and the total severity of traumatic events was calculated.

Premenstrual Symptoms Screening Tool (PSST): The Premenstrual Symptoms Screening Tool (PSST) is a questionnaire that translates DSM-IV criteria for Premenstrual Dysphoric Disorder (PMDD) into a rating scale with degrees of severity (33). The questionnaire assesses the severity of 14 different premenstrual symptoms and degree of interference in five areas of life using a Likert scale (1 to 4, “Not at All” to “Severe”). The PSST stratifies participants into those with no to mild premenstrual symptoms, moderate-to-severe premenstrual symptoms or PMS, and likely PMDD.

Structured Clinical Interview for DSM-IV Disorders (SCID): The Structured Clinical Interview for DSM-IV Disorders (SCID) is a semi-structured interview guide for making DSM-IV diagnoses. The SCID for Axis I assesses for current and lifetime mood disorders, psychotic disorders, substance use disorders, anxiety disorders, and eating disorders through questions that map onto DSM-IV diagnostic criteria (29).

### Participants

Of the four-hundred and nine participants enrolled in the parent study as of June 2020, 18 participants were missing either PSST or CTE data, resulting in a final sample of N = 391 participants used for this analytic cohort. Participants were stratified into 4 groups based on their level of premenstrual distress (as measured by their PSST category) and their history of psychiatric illness as follows: 1) Premenstrual Exacerbation (PME) – those with moderate-to-severe premenstrual symptoms on PSST and either a current mood or anxiety disorder on the SCID or those on medication for a lifetime mood or anxiety disorder; 2) Premenstrual Disorders (PMDs) – those who met “moderate-to-severe PMS” or “PMDD” on the PSST, but did not have current mood/anxiety disorder nor medication treatment for a lifetime mood/anxiety disorder; 3) Controls with psychiatric illness [Psychiatric Controls (PC)] – those with none to mild premenstrual symptoms on the PSST **and** either a current mood/anxiety disorder on the SCID or medication treatment for a lifetime mood/anxiety disorder and 4) Healthy Controls (HC) – those with none to mild premenstrual symptoms, no current mood/anxiety disorder and no medication treatment for a lifetime mood/anxiety disorder.

### Data analysis

Demographics were summarized by group (HC, PC, PMD, PME). Categorical variables were summarized with N (%) and group-level differences were tested using Fisher’s Exact Tests. Analysis of variance (ANOVA) was used to test for group-level differences in the CTE trauma measurements (number of events experienced, severity of events experienced). Adjusted models controlling for demographic variables were conducted to control for baseline differences. For *post-hoc* analysis of any significant ANOVA findings, Tukey’s HSD test was used to test for pairwise comparisons between groups (34). Fisher’s exact test was used to test for univariate differences in type of trauma experienced by group and to evaluate for differences in experience of prepubertal trauma, with prepubertal trauma being defined as trauma experienced before 2 years prior to menarche. Multinomial logistic regression with trauma type as the outcome was used to test for group level differences in types of trauma experienced, adjusting for relevant demographic covariates.

Exploratory correlation analyses assessed the relationship between the total PSST score (sum of all items) and CTE-S trauma measurements (number of events experienced, severity of events experienced) in order to explore the relationship between childhood trauma and premenstrual symptoms using continuous as opposed to categorical groupings.

Exploratory analyses assessed for differences in total PSST scores between the groups using ANOVA, to see if groups with the same categorical level of premenstrual distress (e.g. PME and PMD; PC and HC) experienced the same quantitative level of premenstrual symptoms.

Significance was evaluated at a level of *p* <.05. As this was largely an exploratory hypothesis-generating study, we did not correct for multiple comparisons in order to not miss associations worthy of future study in larger and more rigorous samples. Analyses were completed using R version 3.6.2 (35).

## Results

### Group differences in trauma

#### Sample characteristics

N=217 participants were classified as HC, 102 PC, 34 PMD, and 38 PME. **Table 1** summarizes the participant demographics overall and by group. The groups differed in their distribution of race (*p* = .040), education level (*p* <.001), and income level (*p* <.001). The groups were otherwise similar in terms of age, ethnicity, and employment status.

**Table 1:** Participant characteristics overall and by group.

	Overall (N=391)	HC (N=217)	PC (N=102)	PMD (N=34)	PME (N=38)	P-value
Age						
Mean (SD)	32.7 (4.38)	32.8 (4.15)	32.9 (4.71)	32.6 (4.41)	31.6 (4.72)	.443
Race						
White	292 (74.7%)	169 (77.9%)	74 (72.5%)	24 (70.6%)	25 (65.8%)	**.040**
Black	52 (13.3%)	20 (9.2%)	22 (21.6%)	5 (14.7%)	5 (13.2%)	
Asian or Pacific Islander	25 (6.4%)	16 (7.4%)	2 (2.0%)	3 (8.8%)	4 (10.5%)	
Other	22 (5.6%)	12 (5.5%)	4 (3.9%)	2 (5.9%)	4 (10.5%)	
Ethnicity						
Not Hispanic	362 (92.6%)	201 (92.6%)	96 (94.1%)	33 (97.1%)	32 (84.2%)	.316
Hispanic	26 (6.6%)	15 (6.9%)	5 (4.9%)	1 (2.9%)	5 (13.2%)	
Missing	3 (0.8%)	1 (0.5%)	1 (1.0%)	0 (0%)	1 (2.6%)	
Education Level						
High School or less	17 (4.3%)	7 (3.2%)	8 (7.8%)	1 (2.9%)	1 (2.6%)	**<.001**
Some College or Associates	46 (11.8%)	12 (5.5%)	15 (14.7%)	5 (14.7%)	14 (36.8%)	
College Degree	98 (25.1%)	62 (28.6%)	17 (16.7%)	10 (29.4%)	9 (23.7%)	
At least Some Graduate	229 (58.6%)	135 (62.2%)	62 (60.8%)	18 (52.9%)	14 (36.8%)	
Missing	1 (0.3%)	1 (0.5%)	0 (0%)	0 (0%)	0 (0%)	
Income Level						
$50,000 or less	55 (14.1%)	16 (7.4%)	19 (18.6%)	9 (26.5%)	11 (28.9%)	**<.001**
$51,000 - 100,000	127 (32.5%)	77 (35.5%)	29 (28.4%)	8 (23.5%)	13 (34.2%)	
$100,000 or more	190 (48.6%)	116 (53.5%)	45 (44.1%)	16 (47.1%)	13 (34.2%)	
Missing	19 (4.9%)	8 (3.7%)	9 (8.8%)	1 (2.9%)	1 (2.6%)	
Currently Employed?						
No	66 (16.9%)	31 (14.3%)	23 (22.5%)	5 (14.7%)	7 (18.4%)	.329
Yes	320 (81.8%)	182 (83.9%)	78 (76.5%)	29 (85.3%)	31 (81.6%)	
Missing	5 (1.3%)	4 (1.8%)	1 (1.0%)	0 (0%)	0 (0%)	
Psychotropic Medication Use						
No	312 (79.8%)	217 (100%)	49 (48.0%)	34 (100%)	13 (36.8%)	**<.001**
Yes	72 (18.4%)	0 (0%)	49 (48.0%)	0 (0%)	23 (60.5%)	
Missing	7 (1.8%)	4 (1.8%)	4 (3.9%)	0 (0%)	1 (2.6%)	

Categorical data is represented by N (%) and P-values are for Fisher’s exact test.

HC, Healthy Controls; PC, Psychiatric Controls (Controls with Psychiatric Illness); PMD, premenstrual disorder; PME, premenstrual exacerbation.

Bold values mean significant at a level of p <.05.

#### Number of traumatic events

136 participants (62.7%) in the HC group, 74 participants (72.5%) in PC group, 26 participants (76.5%) in PMD, and 31 participants (81.6%) in PME experienced trauma prior to age 17.

**Figure 1:**
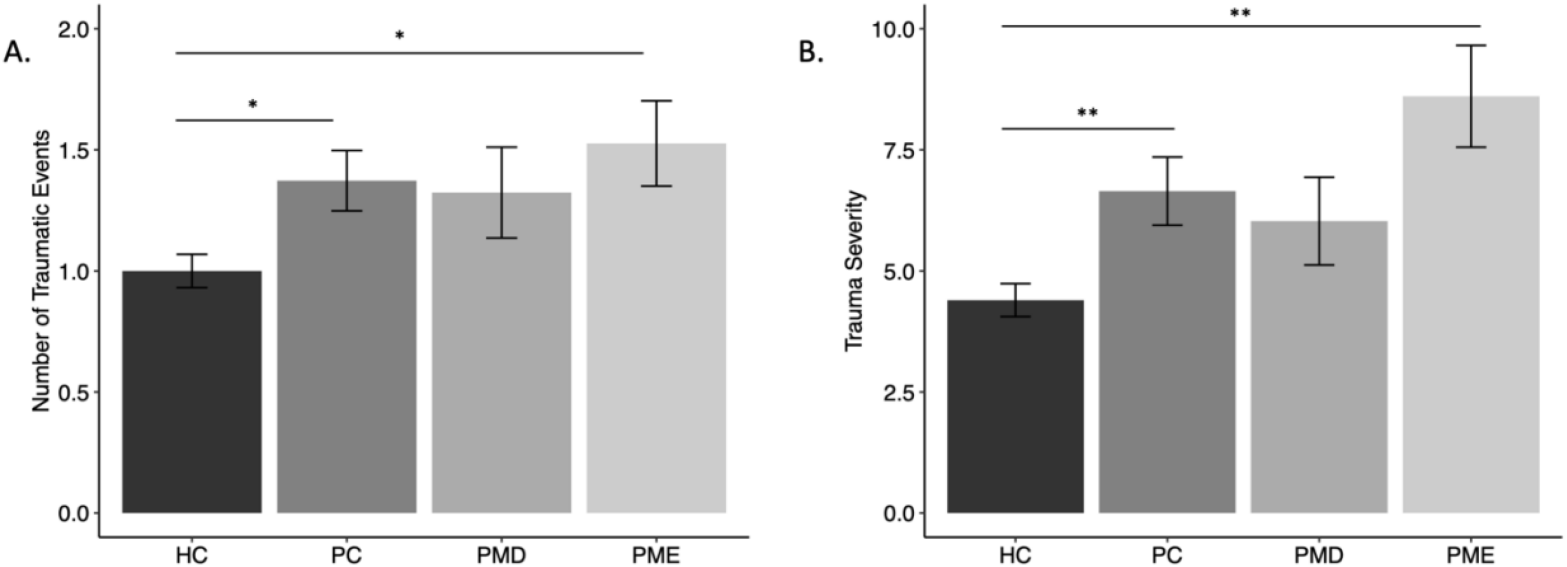
Mean measures of childhood trauma by group. [**(A)** number of traumatic events, **(B)** severity of events] by group. Error bars represent standard error. HC, Healthy Controls; PC, Psychiatric Controls (Controls with Psychiatric Illness); PMD, premenstrual disorder; PME, premenstrual exacerbation. * = p <.05; ** = p <.01.

One-way ANOVA revealed a significant difference in number of traumatic events reported between groups [F(3,387) = 4.46, *p* = .004]. Tukey’s HSD test for multiple comparisons revealed that this was driven by a significant difference between HC and PC (adjusted difference = 0.37, 95% CI = 0.03 – 0.71, *p* = .025) and a significant difference between HC and PME (adjusted difference = 0.53, 95% CI = 0.03 – 1.02, *p* = .033), such that HCs reported fewer traumatic events compared to the PC and PME groups (**Figure 1A**). This difference between groups remained significant after controlling for medication use, race, income, and education [F(3,353) = 5.27, *p* = .001]. No other pairwise comparisons were significantly different (*p*’s >.35).

#### Severity of traumatic events

There was a significant difference in the severity of reported traumatic events among groups (F(3,387) = 7.67, *p* <.001). Tukey’s HSD test for multiple comparisons revealed that differences in severity scores were driven by a significant difference between HC and PC (adjusted difference = 2.25, 95% CI = 0.45 – 4.05, *p* = .007) and a significant difference between HC and PME (adjusted difference = 4.21, 95% CI = 1.58 – 6.84, *p* = .003), such that HCs reported a lower severity of traumatic events compared to the PC and PME groups (**Figure 1B**). This group difference remained significant after controlling for medication use, race, income, and education [F(3,353) = 3.81, *p* = .010]. No other pairwise comparisons were significantly different (*p*’s >.23).

#### Trauma type

**Table 2** depicts the frequency of types of traumas reported within each group. Fisher’s exact test revealed a significant difference between groups, with more participants in the PC and PME groups reporting a history of sexual abuse (*p* = .020) and “other” trauma (*p* = .008), but no significant differences across other trauma types. However, multinomial logistic regression models revealed that there was no significant group difference in experience of sexual abuse once controlling for medication use, race, income, and education (*p*s >.15), but that those who earned higher income had lower odds of experiencing sexual abuse in childhood compared to those making $50,000 or less (**Table 3**). Participants who earned between $50,000 and $100,000 (OR = 0.39, 95% CI = 0.15 - 0.99, p = .047) and those making $100,000 or more (OR = 0.20, 95% CI = 0.07 - 0.57, p = .003) had lower odds of experiencing sexual abuse in comparison to those making $50,000 or less. Additionally, when controlling for medication use, race, income, and education, there was no longer a group-level difference or any significant demographic predictors of experiencing other types of trauma (**Table 3**).

**Table 2:** Types of childhood traumas experienced by group.

	HC (N=217)	PC (N=102)	PMD (N=34)	PME (N=38)	P-value
Trauma: Death					
No	142 (65.4%)	66 (64.7%)	21 (61.8%)	23 (60.5%)	.903
Yes	74 (34.1%)	36 (35.3%)	13 (38.2%)	15 (39.5%)	
Missing	1 (0.5%)	0 (0%)	0 (0%)	0 (0%)	
Trauma: Divorce					
No	164 (75.6%)	67 (65.7%)	22 (64.7%)	27 (71.1%)	.199
Yes	51 (23.5%)	34 (33.3%)	12 (35.3%)	11 (28.9%)	
Missing	2 (0.9%)	1 (1.0%)	0 (0%)	0 (0%)	
Trauma: Sexual Abuse					
No	195 (89.9%)	83 (81.4%)	28 (82.4%)	27 (71.1%)	**.020**
Yes	21 (9.7%)	18 (17.6%)	5 (14.7%)	10 (26.3%)	
Missing	1 (0.5%)	1 (1.0%)	1 (2.9%)	1 (2.6%)	
Trauma: Other Violence					
No	201 (92.6%)	94 (92.2%)	30 (88.2%)	32 (84.2%)	.266
Yes	16 (7.4%)	7 (6.9%)	4 (11.8%)	6 (15.8%)	
Missing	0 (0%)	1 (1.0%)	0 (0%)	0 (0%)	
Trauma: Severe Illness					
No	201 (92.6%)	91 (89.2%)	32 (94.1%)	35 (92.1%)	.755
Yes	16 (7.4%)	11 (10.8%)	2 (5.9%)	3 (7.9%)	
Trauma: Other					
No	178 (82.0%)	68 (66.7%)	25 (73.5%)	24 (63.2%)	**.008**
Yes	39 (18.0%)	34 (33.3%)	9 (26.5%)	13 (34.2%)	
Missing	0 (0%)	0 (0%)	0 (0%)	1 (2.6%)	

Categorical data is represented by N (%) and P-values are for Fisher’s exact test.

HC, Healthy Controls; PC, Psychiatric Controls (Controls with Psychiatric Illness); PMD, premenstrual disorder; PME, premenstrual exacerbation.

Bold values mean significant at a level of p <.05.

**Table 3:** Multinomial logistic regression for trauma type outcomes (sexual abuse and other types of trauma).

	Odds Ratio	95% CI	p-value
Sexual Abuse			
**Group**			
HC	1.00 (ref)		
PC	1.05	0.39 – 2.62	.919
PMD	1.12	0.32 – 3.31	.845
PME	1.05	0.28 – 3.51	.939
Medication Use			
No	1.00 (ref)		
Yes	2.29	0.86 - 6.43	.103
**Income**			
$50,000 or less	1.00 (ref)		
**$51,000 - 100,000**	**0.39**	**0.15 – 0.99**	**.047**
**$100,000 or more**	**0.20**	**0.07 – 0.57**	**.003**
Education Level			
High School or less	1.00 (ref)		
Some College or Associates	1.48	0.40 – 5.98	.561
College Degree	0.81	0.19 – 3.61	.775
At least Some Graduate	0.63	0.17 – 2.59	.501
Race			
White	1.00 (ref)		
Black	0.83	0.29 – 2.19	.714
Asian or Pacific Islander	1.53	0.39 – 4.83	.497
Other	0.75	0.15 – 2.66	.683
Other Violence			
Group			
HC	1.00 (ref)		
PC	1.98	0.98 – 3.93	.054
PMD	1.64	0.66 – 3.81	.264
PME	2.13	0.77 – 5.63	.135
Medication Use			
No	1.00 (ref)		
Yes	1.14	0.53 - 2.49	.739
Income			
$50,000 or less	1.00 (ref)		
$51,000 - 100,000	0.74	0.32 – 1.76	.491
$100,000 or more	0.74	0.31 – 1.84	.501
Education Level			
High School or less	1.00 (ref)		
Some College or Associates	1.01	0.29 – 3.66	.986
College Degree	0.69	0.20 – 2.61	.579
At least Some Graduate	0.75	0.23 – 2.65	.645
Race			
White	1.00 (ref)		
Black	1.14	0.48 – 2.58	.762
Asian or Pacific Islander	0.99	0.31 – 2.70	.987
Other	1.60	0.55 – 4.26	.359

Bold values mean significant at a level of p <.05.

#### Prepubertal timing of trauma

61 participants (28.1%) in the HC group, 30 participants (29.4%) in PC group, 17 participants (50%) in PMD, and 16 participants (42.1%) in PME experienced prepubertal trauma. Fisher’s Exact Test showed that there was not a significant difference between groups in number of individuals with prepubertal trauma exposure (*p* = .125).

### Association between trauma measures and premenstrual symptoms in the total sample

#### Number of traumatic events

**Figure 2:**
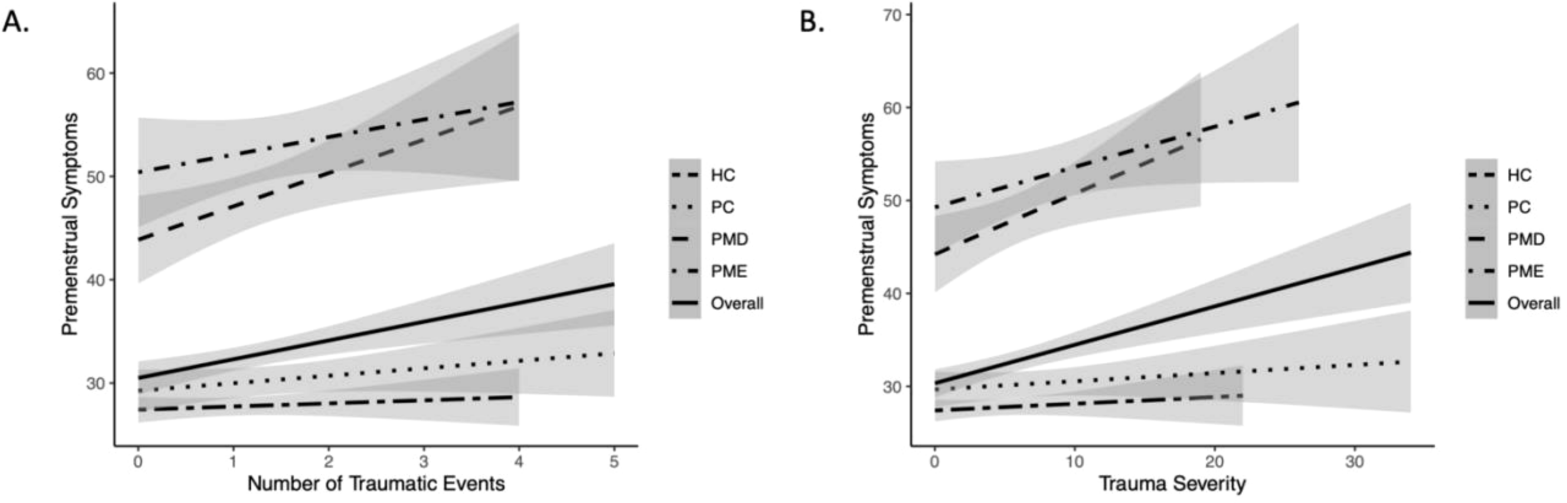
Association between measures of childhood trauma and premenstrual symptoms overall and within each group. [**(A)** number of traumatic events, **(B)** severity of events] and premenstrual symptoms (measured by PSST) by group. HC, Healthy Controls; PC, Psychiatric Controls (Controls with Psychiatric Illness); PMD, premenstrual disorder; PME, premenstrual exacerbation.

Across groups, there was a weak but significant positive correlation between number of childhood traumatic events and total PSST score (R = .18, *p* <.001), see **Figure 2A**. Upon testing for correlations within each group, the association was significant within the PMD group (R = .42, *p* = .013), but not the PME (R = .20, *p* = .234), HC (R = .05, *p* = .495), or PC groups (R = .13, *p* = .181).

#### Severity of traumatic events

Across groups, there was a weak but significant positive correlation between the severity of childhood traumatic events and total PSST score (R = .22, p <.001), see **Figure 2B**.

Correlation analyses within each group revealed a significant association within the PMD group (R = .41, *p* = .015), but not the PME (R = .30, *p* = .066), HC (R = .05, *p* = .422), or PC groups (R = .09, *p* = .348).

### Group differences in premenstrual symptoms

Exploratory analyses were run to assess whether premenstrual symptoms (as measured by the total PSST score) differed between the PME and PMD groups (**Table 4**). ANOVA examining PSST scores revealed a significant difference by group (F(3,387) = 177.9, p <.001) *Post-hoc* analyses revealed a significant difference between the PME and PMD groups (adjusted difference = 4.85, 95% CI = 0.28 – 9.43, *p* = .033), such that those in the PME group experienced higher levels of premenstrual symptoms compared to the PMD group, but there was no difference between the HC and PC groups (adjusted difference = 1.95, 95% CI = - 0.38 – 4.27, *p* = .136). All other pairwise comparisons (PMD-HC, PMD-PC, PME-HC PME-PC) were significantly different from each other (*p* <.001). After controlling for medication use, race, income, and education level, there was a significant group effect (F(3,353) = 175.0, *p* <.001), but no longer a significant difference between the PME and PMD groups in *post-hoc* analyses (adjusted difference = 4.10, 95% CI = - 0.43 – 8.64, *p* = .092).

**Table 4:** Premenstrual symptom burden by group.

	HC (N=217)	PC (N=102)	PMD (N=34)	PME (N=38)	P-value
**Premenstrual Symptoms Screening Tool Sum** Mean (SD)	27.7 (6.68)	29.7 (8.12)	48.1 (8.35)	53.0 (9.32)	<.001

HC, Healthy Controls; PC, Psychiatric Controls (Controls with Psychiatric Illness); PMD, premenstrual disorder; PME, premenstrual exacerbation.

## Discussion

The current study examined the relationships among PMDs, PME, psychiatric illness, and childhood trauma experiences. We found that the experience of childhood trauma (number of traumatic events and severity of traumatic events) differed by group; participants with PME, and participants with a current or lifetime mood/anxiety disorder (PC), had a significantly higher number of traumatic events and greater severity of trauma compared to HCs. It is notable, however, that our study did not find significant differences in exposure to specific types of traumatic events after confounding for variables. A childhood history of sexual abuse, for example, have been previously associated with PMDs (18, 36, 37). This discrepancy is likely a result of differences in the questionnaires used across studies. For example, most data evaluating early childhood trauma and adversity as it relates to PMDs have utilized the ACE Questionnaire (ACE-Q) (38) or the Childhood Trauma Questionnaire (CTQ) scales. Indeed, both the ACE-Q and CTQ specifically ask questions about emotional, physical, and sexual abuse and/or neglect. Physical and emotional abuse, as well as emotional neglect, have all been specifically associated with the development of PMDs (7, 39, 40). Since the CTE-S does not ask about emotional abuse or neglect, however, our results may have failed to show any differences in trauma type in the PMDs group for this reason. Additionally, sexual abuse has been associated with higher rates of developing PMDD, but due to the limited number of participants who met criteria for PMDD (n=6) in this study, we were underpowered to evaluate this difference. It is interesting, however, that the specific types of traumatic events measured by the CTE-S (e.g., divorce, death of family/friend, illness/injury, or being a victim of violence) were not associated with PMD status but do appear to be associated with the risk for psychiatric illness more generally. Regarding premenstrual symptom severity, we found a weak but significant correlation between childhood trauma (number of traumatic events and severity of trauma experience) and higher premenstrual symptom burden. Within-group analysis suggested that this association was driven by correlations within the PMD group but not the other groups. Indeed, this finding is supported by other studies, which also found a correlation between the frequency and intensity of traumatic experiences and the corresponding severity of premenstrual symptoms (16, 18, 23). Although we did not detect a significant pairwise difference between PMDs and HCs in premenstrual symptom severity, it is worth highlighting that the data (see **Figure 1**) show a similar pattern for both PMD and PME patients. In both cases the frequency and severity of trauma were higher relative to HCs. Future studies with larger sample sizes, particularly among those with PMD, may observe statistically significant effects. We also found that those with PME experienced quantitatively higher levels of premenstrual symptoms on the PSST compared to the PMD group, despite both groups scoring in the moderate to severe range on PSST. However, after controlling for medication use, race, income, and education level, the initial difference in PSST scores between the PME and PMD groups was no longer statistically significant in *post-hoc* analyses. Together, this suggests that socioeconomic and demographic factors may partially account for the observed differences in elevated premenstrual symptom severity observed in the PME group. Another potential explanation is that the PME group may have represented a more psychiatrically ill population (as indicated by the increased presence of medication use). In this case those with PME may be experiencing ongoing symptoms of anxiety and depression throughout the month that (by definition) that worsen in the premenstrual period. Particularly, if the psychiatric symptoms experienced outside of the premenstrual time are already moderate to severe, it may be that the premenstrual worsening represents a time of additional suffering on an already stressed individual. Additionally, it is known that individuals experiencing depression have a subjectively enhanced experience of both physical and psychic suffering, which may further intensify the experience of physical and emotional premenstrual symptoms (41–43). To date, there are no known studies that have evaluated (qualitatively nor quantitatively) the symptom trajectory, characteristics, or severity of symptoms among individuals with PME across the menstrual cycle. Larger studies will be needed to determine whether there are differences in the subjective experience of premenstrual symptoms based on primary diagnosis and sociodemographic factors. It will be interesting to expand on the current findings to elucidate if higher PSST scores are associated with worse baseline mood or anxiety symptoms, or if PSST scores are independent of overall psychiatric severity outside of the premenstrual window.

Together, our results show that while PME and psychiatric diagnosis were associated with more frequent and more severe trauma exposure than in healthy controls, PMDs categorically were not associated with greater trauma exposure. Instead, within the PMD group, the number and severity of traumatic events was associated with greater premenstrual symptom load, which was not true in the other groups. Thus, it appears that trauma exposure increases risk for psychiatric diagnosis and PME generally, but among those with PMDs, it is the premenstrual symptoms that are more severe with greater exposure to childhood trauma. These results extend the expanding realm of research concerning the role of childhood adversity in reproductive affective disorders. Indeed, a history of trauma has been linked to most reproductive psychiatric illnesses, including PMDs, postpartum depression, and perimenopausal depression (44, 45). However, it is unknown the mechanisms by which childhood trauma would increase risk for reproductive affective disorders, which are characterized by sensitivity to gonadal hormone fluctuations. Recent data have suggested that the interplay between the hypothalamic-pituitary-adrenal (HPA) and hypothalamic-pituitary-gonadal (HPG) axes, particularly the modulation of the HPA axis by gonadal hormones such as estrogen and progesterone and their neuroactive steroid metabolites, may underlie the elevated prevalence of mood disorders observed among women (45–48). Particularly relevant to women, who experience higher levels of childhood adversity than males, early life adversity may prime the HPA axis, resulting in more sensitivity to the natural gonadal hormone fluctuations across the reproductive life span. Intriguingly, recent research has demonstrated distinct profiles in cortisol function across the menstrual cycle among individuals with PMS and PMDD. In a study examining the intersection of childhood adversity and PMDs, women with PMDD and a history of abuse had greater premenstrual mood symptoms and higher cortisol levels than those with no abuse history, as they transitioned from the gonadal hormone milieu of the follicular phase to the luteal phase of the menstrual cycle (15). To date, there have been no specific investigation into HPA-HPG axis function in PME; future studies might discern whether the HPA axis profiles across the menstrual cycle among individuals with PME resemble those of individuals with PMS, PMDD, or MDD. In addition, gonadal hormone interaction with the serotonergic and GABAergic systems have been an important focus in exploring potential biological contributors to PMD risk (10, 15).

It’s important to consider some important limitations when interpreting our findings. Firstly, our study assessed childhood trauma based on participants’ retrospective self-reports, which naturally introduces a potential for recall bias. In addition to the challenge of recall bias, retrospective assessments limit the evaluation of any confounding variables that may have affected the outcome assessed. For example, the PSST asks retrospectively about premenstrual physical and mood symptoms, but we did not prospectively assess mood symptoms across the menstrual cycle. Additionally, while our study created groupings based on current PMD and psychiatric status to examine group differences in past ACEs, we acknowledge that the reverse approach could also be informative, i.e. grouping by ACE status (49, 50); however, we employed ANOVA evaluation to specifically observe differences across groups as we could not evaluate directionality in this retrospective analysis.

Although our study relied on validated measures to evaluate early life trauma, the CTE-S is not as widely used as the ACE-Q or CTQ, limiting generalizability of this study, and does not contain some of the key trauma domains (e.g. physical or emotional abuse/neglect) that have been previously associated with PMDs. The lack of information about emotional abuse and neglect may have limited our findings and ability to discern differences between our participant groups. Additionally, due to large differences in our sample sizes between groups and the relatively small number of participants with PMDD, our study may have been underpowered to recognize more subtle trends among groups. Further, the CTE-S only assesses trauma experienced in childhood and adolescence, and thus we were not able to account for the potential impact of stress or trauma experienced post-adolescence on PMD/PME risk.

Despite these limitations, our research contributes to an emerging field that confirms connections among childhood trauma, premenstrual symptoms, and psychiatric disorders. Our study uniquely focuses on evaluating the impact of trauma specifically on premenstrual symptoms and particularly among individuals with PME, a condition that has received insufficient attention in research and clinical practice. Our findings reveal that individuals with PME experience more severe premenstrual symptoms compared to those with PMD, underscoring the importance of further investigation, clinical assessment, and intervention for this specific patient group. Future studies focused on exploring the link between trauma and PME may provide valuable insights into the underlying mechanisms connecting early life trauma or adversity to reproductive psychiatric disorders. Additionally, exploring the hormonal and neurobiological pathways involved in PME (particularly differences in the HPA and HPG axes), in comparison to those with mood disorders alone or in comparison to PMDs, may help elucidate the biological bases of these illnesses and fuel investigations to identify potential biological targets for intervention.

## Data Availability

The raw data supporting the conclusions of this article will be made available by the authors, without undue reservation.
